# In silico investigation on sensing of tyramine by boron and silicon doped C_60_ fullerenes

**DOI:** 10.1038/s41598-023-49414-5

**Published:** 2023-12-14

**Authors:** S. Pattanaik, A. K. Vishwkarma, T. Yadav, E. Shakerzadeh, D. Sahu, S. Chakroborty, P. K. Tripathi, E. A. Zereffa, J. Malviya, A. Barik, S. K. Sarankar, P. Sharma, V. J. Upadhye, S. Wagadre

**Affiliations:** 1 Sri Satya Sai University of Technology and Medical Sciences, Sehore, Bhopal, M.P. India; 2 Department of Physics, Institute of Science, Banaras Hindu University, Varanasi, India; 3 Department of Basic Sciences, IITM, IES University, Bhopal, M.P. India; 4 Chemistry Department, Faculty of Science, Shahid Chamran University of Ahvaz, Ahvaz, Iran; 5 School of Applied Sciences, Centurion University of Technology and Management, Bhubaneswar, Odisha India; 6 Department of Physics, Sharda University, Greater Noida, U.P. India; 7 School of Applied Natural Science, Department of Applied Chemistry, Adama Science and Technology University, Adama, Ethiopia; 8 Department of Life Sciences and Biological Sciences, IES University, Bhopal, M.P. India; 9 CIPET: Institute of Petrochemicals Technology [IPT], Bhubaneswar, Odisha India; 10 Faculty of Pharmacy, Mansarovar Global University, Sehore, M.P. 466111 India; 11 Department of Pharmacy, Barkatullah University, Bhopal, India; 12 Departmentt of Microbiology, Parul Institute of Applied Sciences (PIAS), Parul University, PO Limda, Tal Waghodia, 391760 Vadodara, Gujarat India

**Keywords:** Chemistry, Materials science, Nanoscience and technology, Physics

## Abstract

The present communication deals with the adsorption of tyramine neurotransmitter over the surface of pristine, Boron (B) and Silicon (Si) doped fullerenes. Density functional theory (DFT) calculations have been used to investigate tyramine adsorption on the surface of fullerenes in terms of stability, shape, work function, electronic characteristics, and density of state spectra. The most favourable adsorption configurations for tyramine have been computed to have adsorption energies of − 1.486, − 30.889, and − 31.166 kcal/mol, respectively whereas for the rest three configurations, it has been computed to be − 0.991, − 6.999, and − 8.796 kcal/mol, respectively. The band gaps for all six configurations are computed to be 2.68, 2.67, 2.06, 2.17, 2.07, and 2.14 eV, respectively. The band gap of pristine, B and Si doped fullerenes shows changes in their band gaps after adsorption of tyramine neurotransmitters. However, the change in band gaps reveals more in B doped fullerene rather than pristine and Si doped fullerenes. The change in band gaps of B and Si doped fullerenes leads a change in the electrical conductivity which helps to detect tyramine. Furthermore, natural bond orbital (NBO) computations demonstrated a net charge transfer of 0.006, 0.394, and 0.257*e* from tynamine to pristine, B and Si doped fullerenes.

## Introduction

The remarkable properties of organic nano-structured materials viz zero-dimensional nano-cages, one-dimensional nano-sheets, two-dimensional nano-tubes, or three-dimensional diamond and others have been used extensively for modeling biosensors and drug carriers^1–17^. Numerous  organic nano-materials based biosensors have been exhaustively predicted and imprisoned to detect a number of bio-relevant drugs or molecules including proteins, DNA, and neurotransmitters etc.^18–27^. A number of investigations show that fullerenes cages are used in the development of biosensors for biomolecules and drug delivery systems because of their high sensitivity to drug molecules, non-toxicity, unique spherical shape, and structural stability^28,29^. When silicon or boron atoms are added, fullerenes offer a number of benefits that make them suitable for use in sensing applications. The electrical and optical characteristics of fullerenes can be changed by doping them with silicon and boron, which increases their sensitivity and selectivity for specific biomolecules. Dopant atoms change the electronic band structure of the fullerene by adding new energy levels, which alters the material's optical sensitivity and conductivity. These altered characteristics enable the detection and identification of many chemical and biological entities. Furthermore, boron-doped carbon nanomaterials may act as p-type conductors with electronic holes, whereas silicon-doped nanomaterials show metal conductivity. The charge distribution of the fullerene system can also be altered by doping it with silicon and boron. This alters the band gap, which is important for the ability of systems to absorb and function as sensors^30–35^.

Tyramine acts as a neurotransmitter and is a vital trace biogenic amine^36^. Besides, in various moods, emotion, cognition, and attention-related human illnesses, trace biogenic amines are key contributors. The mammalian brain and peripheral nerve tissues release tyramine^37,38^. Fish, meat, fruits, cheese, soy products, and wine are among a variety of raw and fermented foods that contain excessive quantities of tyramine^39^. Tyramine rich food is detrimental to human health when consumed in large amounts because it causes symptoms like flushing, rash, hypertonia, vomiting, heart palpitations, and tachycardia. Additionally, it raises risky blood pressure and raises the likelihood of severe migraine headaches^40^. To carry out early diagnosis of the aforementioned disorders, it is therefore extremely important to identify a suitable sensing device to detect tyramine in biological components and food products that are rich in tyramine with enough sensitivity.

Our literature survey reveals that there have been a number of studies including experimental and theoretical dealing with the modeling of biosensors for neurotransmitters such as dopamine and various kinds of other bio-relevant molecules by using TiO_2_, CdSe, fullerenes, carbon nanotube (CNTs), Au/ZnO, In2O3 and other organic based nano-structured materials as well^18–27,41–47^. In order to develop a suitable biosensor for the detection of dopamine neurotransmitter in biological circumstances, Yeh and co-workers^2^ undertook an in silico analysis on the interaction of dopamine neurotransmitter with single-walled carbon nanotubes including pristine as well as boron and nitrogen doped nanotubes. The interaction of dopamine neurotransmitter with the TiO_2_ was explored by Urdaneta and co-workers^42^. In their study, they reported a strong adsorption of dopamine with TiO_2_. Additionally, it has been recommended that fullerenes be functionalized to enhance charge transfer, which makes fullerenes useful in the development of exceptionally sensitive biosensors^1^. The pristine B_12_N_12_ and Cu-decorated nano-cages have been proposed as sensors detecting biogenic amines, especially Putrescine^48^. It had been anticipated that the alkali and alkaline earth metals contained in B_36_N_36_ nano-cages could be used as sensors to identify the anticancer drug 5-fluorouracil^49^. The application of BN-fullerene as a biosensor for identifying the -glucose molecule has been reported by Hernández et al.^6^. Graphene quantum dots doped with boron and sulfur have been shown to detect the neurotransmitter dopamine^50^.

Interactions of various nanotube with biomolecules have also been reported by Mohammadi and his group^51–60^. Mohammadi and coworkers explored the binding of fluorouracil with boron nitride (BN), Al-doped boron nitride (Al-BN), and BC_2_N nanotubes where they reported that fluorouracil adsorbs more effectively with Al-doped boron nitride nanotube than boron nitride (BN) and BC_2_N nanotubes^51^. The same group also investigated the effect of Al and Ga atoms doping on the adsorption process of H_2_SiCl_2_ over the surface of boron nitride nanotube. They predicted that Ga doped BN nanotube shows higher affinity towards H_2_SiCl_2_^52^. He, Ne, Ar, and Kr rare gases interaction with the pristine, Al and Ga atoms doped BN nanotubes using various functionals and basis set in order to devolve a suitable sensor for these gases^53^. In this study, the authors reported that Al and Ga atoms doped BN nanotubes are better than pristine BN nanotube for the sensing application. The sensing of Nobel gases by pristine and decorated AlN and SiC nanotubes were investigated using DFT calculations and it was found that Ga and Ge-doped nanotubes are potential candidates for sensing of the Nobel gases^54^. Mohammadi and coworkers performed a theoretical investigation on the interaction of cysteine on pristine BN, Al-doped BN and carbon BN nanotubes to understand various interactions involved between cysteine and above cited nanotubes^55^. The adsorption of Serine amino acid over pristine fullerene (C_60_), Si and Ge doped C_60_ fullerenes were analyzed at B3LYP-D3, ωB97XD, and M062X functionals^56^. Furthermore, Chlorofluoromethane adsorption process over pristine and Ge doped SiC nanotubes was investigated in order to explore the potential of these nanotubes in development of biosensor^57^. Besides, the interactions of other bimolecular species with nanomaterials were studies and discussed in detail by Mohammadi and coworkers^58–60^. Ganji and coworkers^61^ recently reported the interaction between tyrosine and g-Mg_3_N_2_ monolayer using DFT simulations where they found that Mg sites are favorable for the significant interaction between tyrosine and g-Mg_3_N_2_ monolayer. The authors also ensured the stability of tyrosine@g-Mg_3_N_2_ nano-hybrid by molecular dynamics (MD) simulations. Interaction of glycine and metal organic frameworks (MOFs) was analyzed by DFT and the corresponding adsorption energy was predicted − 45.251 kcal/mol^62^. The binding mechanism of Au_32_ and C_60_ cages with 5-Fluorouracil drug was understood by ab initio computations and effect of aqueous medium was also investigated^63^. Moreover, a theoretical investigation dealing with the functionalization of graphene and ZnO monolayers with mercaptopurine at aqueous media was reported by Aghahosseini and coworkers^64^. Comprehensive studies using dispersion-corrected density functional theory (DFT) simulations were conducted to investigate the noncovalent functionalization of chitosan with the drug teriflunomide by Tanreh and coworkers^65^. Recently, we have also reported histamine sensing by B and Si doped fullerenes^66^.

The main focus of the ongoing study is the comprehensive in silico investigation of the interactions of pristine, B and Si doped fullerenes with tyramine neurotransmitter to explore their possible application in developing a suitable biosensor for tyramine. We have previously investigated various probable conformers, geometrical aspects, vibrational dynamics, and NBO analysis of tyramine neurotransmitter^36^. Herein, we are interested to explore the adsorption of tyramine neurotransmitter over the surface of pristine, B and Si doped C_60_ fullerenes to model a suitable sensor to detect tyramine. The findings of the present investigation could render it possible to develop cost effective biosensors that are suitable for detecting tyramine in biological substances and food products.

## Computational details

Theoretical calculations for the optimization of tyramine, pristine, doped fullerenes and fullerene based tyramine nano-hybrid were carried out at B3LYP/6-31G(d,p) level available in Gaussian16 software^67^. Moreover, the harmonic vibrational frequencies were also computed at same level of theory to ensure the stability of the electronic structures. The functional B3LYP stands for the combination of several exchange–correlation functionals which is frequently employed functional in density functional theory (DFT) calculations due to its relatively good performance across variety of molecular systems^56,67^. Conversely, the 6-31G(d,p) basis set is appropriate for a variety of molecular systems because it finds a balance between computational cost and accuracy. It is often applied to medium- to large-scale systems including organic molecules where a respectable degree of accuracy is required without incurring undue computational costs. The inclusion of diffuse and polarization functions help to capture electron distribution more accurately, especially in regions where standard basis sets might struggle^56,67^. The adsorption energies in the most favorable structures of tyramine@C_60_, tyramine@C_59_B and tyramine@C_59_Si were computed as follows:

1 $$E_{ads} = E_{Tyr@Ful} {-}(E_{Ful} + \, E_{Tyr} ) + {\text{BSSE}}$$

where *E*_*Tyr@Ful*_ denotes the total energy of tyramine@C_60_, tyramine@C_59_B and tyramine@C_59_Si systems, *E*_*Ful*_ is the total energy of fullerene (*i.e.* C_60_, C_59_B or C_59_Si) and *E*_*Tyr*_ is the total energy of the optimized tyramine. For the adsorption energy, the counterpoise approach was implemented to correct the basis set superposition error (BSSE). Negative adsorption energy values infer that adsorption process of tyramine over the surface of the corresponding fullerenes is thermodynamically favorable. Total charge transfer between the tyramine and pristine and doped fullerenes has been computed by natural bond orbital (NBO) calculations. Besides, the Gibbs free energy^67^ is computed by following relation-

2 $$\triangle{\text{G}}^{0} \left( {{298}} \right) = \sum {\text{final}}(\varepsilon_{0} + G_{corr} ) - \sum {\text{Inial}}(\varepsilon_{0} + G_{corr} )$$

Here, $(\varepsilon_{0} + G_{corr} )$ is the total sum of electronic and thermal free energies**.**

## Results and discussion

**Figure 1:**
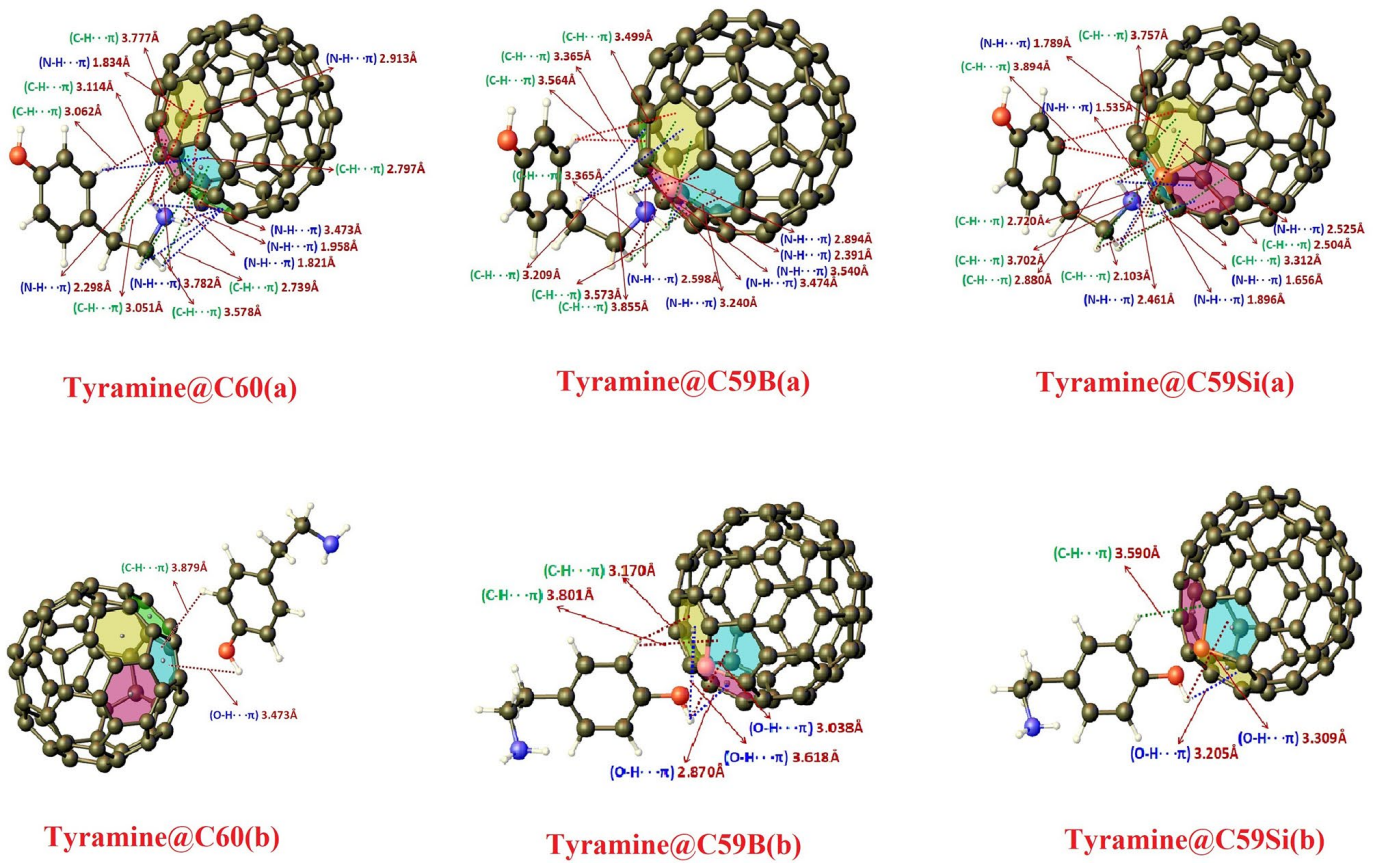
Optimized structures of Tyramine@C_60_, Tyramine@C_59_B and Tyramine@C_59_Si for the various configurations.

A probable number of low lying energy conformers of tyramine neurotransmitter have already been predicted in our previously reported study where we also explored the interaction of tyramine with hydrochloride (HCl) and reported its vibrational dynamics^36^. Now we are exploring the interaction of the most stable conformer of tyramine neurotransmitter as reported in our earlier study with pristine, B and Si doped fullerenes as well. For this reason, the most stable conformer of tyramine has been placed at various positions above the surface of pristine and doped fullerenes and optimization for tyramine@fullerenes nano-hybrid system was performed in order to find out possible geometrical configurations of tyramine@C_60_, tyramine@C_59_B and tyramine@C_59_Si nano-hybrids in gas phase. Theoretical calculations consequences six possible stable nono-hybrides formed though interaction of tyramine with pristine, B and Si doped fullerenes which have been depicted in Fig. 1. From Fig. 1, it infers that the interaction of tyramine with fullerenes produces significant changes in geometrical structures of fullerenes. However, this change reveals more in Si-doped fullerene than pristine and B doped fullerenes. We have also computed the distance between different atoms of tyramine and C_60_/C_59_B/C_59_Si fullerenes which infer good consent with earlier predicted values^10^.

It can be seen in Fig. 1 that the adsorption of tyramine on the surfaces of pristine, B and Si doped fullerenes is mainly governed by C–H···π, N–H···π and O–H···π interactions which are non-covalent in nature^57,69^. All possible intermolecular contacts between tyramine and pristine, B and Si doped fullerenes have been shown in Fig. 1. Moreover, the appropriate magnitude of adsorption energy is key factor while devolvement of bio-sensors. Thus, we have computed the adsorption energy for all six possible configurations of tyramine@C_60_, tyramine@C_59_B and tyramine@C_59_Si nano-hybrid systems by using Eq. (1) and these values are listed in Table 1. The interaction of ethyleamine side chain of tyramine with surface of pristine C_60_ fullerene results − 1.486 kcal/mol (Without BSSE) adsorption energy. However, the same value shows a change in its magnitude by 0.495 kcal/mol upon interaction with OH containing ring part. This change in the adsorption energy has been occurred due to less C-H···π and absence of N–H···π interactions involved between OH containing ring part and surface of pristine C_60_ fullerene in comparison to ethylamine side chain. The value of adsorption energy clearly reveals that adsorption of tyramine over the surface of pristine fullerene is more extensive from ethyleamine side chain part and the adsorption process is exothermic physisorption in nature^70^. A net charge transfer of 0.006*e* from tyramine to pristine fullerene has been computed with the help of NBO calculation*.* Furthermore, our calculations for adsorption energy indicate that the pristine C_60_ fullerene do not have good adsorption capability to adsorb tyramine neurotransmitter.

**Table 1:** Adsorption energies for various configurations.

Configurations	E_ads_ (kcal/mol)	BSSE (kcal/mol)
Tyramine@C_60_(a)	− 1.486	2.236
Tyramine@C_60_(b)	− 0.991	1.523
Tyramine@C_59_B(a)	− 30.889	5.385
Tyramine@C_59_B(b)	− 6.999	4.438
Tyramine@C_59_Si(a)	− 31.166	5.636
Tyramine@C_59_Si(b)	− 8.796	4.892

To improve the adsorption capacity of pristine C_60_ fullerene towards tyramine, chemical modifications caused by doping B and Si atoms with the substitution of carbon atoms have been made. The doping of aforesaid atoms improves the C–H···π and N–H···π contacts which plays a crucial role in the adsorption of tyramine over C_59_B and C_59_Si fullerenes. In addition to C–H···π and N–H···π contacts, the doping of these two atoms expedite to the emergence of dative bond (N–B) and covalent bond between tyramine and surface of C_59_B and C_59_Si fullerenes in their most favorable adsorption configurations *i.e.*tyramine@C_59_B(a) and tyramine@C_59_Si(a) respectively^2^. The C–H···π and N–H···π contacts along with presence of dative (N-B) and covalent bonds between tyramine and C_59_B and C_59_Si fullerenes surfaces have a significant impact on the stability of tyramine@C_59_B(a) and tyramine@C_59_Si(a) nano-hybrid systems. These causes more negative adsorption energy. The magnitude of adsorption energy after chemical modification are computed to be − 30.889, and − 31.166 kcal/mol in their most favorable configurations *i.e.* for tyramine@C_59_B(a) and tyramine@C_59_Si(a) respectively. These two values are without involving BSSE correction term and including BSSE these comes to be − 25.504 and − 25.53 kcal/mol respectively. Besides, the magnitude of adsorption energies infers a chemical adsorption process when ethyamine side chain interacts with C_59_B and C_59_Si fullerenes surfaces^70^. However, in rest two configurations of tyramine@C_59_B and tyramine@C_59_Si, the adsorption energies are − 6.999 and − 8.796 kcal/mol respectively. The above discussion clearly reveals a significant role of doping of B and Si atoms in the increased adsorption capacity towards tyramine. Thus, we can conclude that B and Si atoms doped fullerenes are more sensitive towards tyramine rather than pristine C_60_ fullerene. Moreover, the NBO calculations show that 0.394 and 0.257*e* charge takes place from tyramine to B and Si atoms doped fullerenes in their most favorable configurations respectively.

The development of bio-sensors strongly depends upon the highest occupied molecular orbital (HOMO) and lowest unoccupied molecular orbital (LUMO) energy gap (*E*_*g*_ = $\varepsilon_{LUMO}$ − $\varepsilon_{HOMO}$) analysis. Consequently, the variation in band gap *i.e. E*_*g*_ values for C_60_, C_59_B and C_59_Si fullerenes before and after adsorption of tyramine have been computed for all six configurations and listed in Table 2. The electrical conductivity (*σ*) of tyramine@fullerene nano-hybrid systems at a particular temperature can be estimated in accordance to the following relation^71^

3 $$\sigma \propto e^{{\left( {\frac{{ - E_{g} }}{2KT}} \right)}}$$

where *K* and *T* stand are the Boltzmann constant and temperature respectively. This equation illustrates that elevated electrical conductivity results from a lower energy gap (*E*_*g*_). The electrical conductivity is converted into electrical signals for the purpose of detecting drugs. Before adsorption of tyramine, the energy gap (*E*_*g*_) for pristine, B and Si doped C_60_ fullerenes has been computed to be 2.75, 2.44, and 2.17 eV, respectively. However, tyramine adsorption over the surfaces of these fullerenes lowers the energy gap and it becomes 2.68, 2.06, and 2.07 eV, respectively. Here, we can see that the change in energy gap for pristine C_60_ fullerene is not significant whereas it is noteworthy in case of tyramine@C_59_B(a) and tyramine@C_59_Si(a) nano-hybrid systems. Overall, the adsorption energy values and change in band gap suggests that B and Si doped fullerenes are more suitable to detect tyramine than pristine C_60_ fullerene.

**Table 2:** Calculated HOMO energy (E_H_), LUMO energy (E_L_), HOMO–LUMO energy gap (E_g_), Fermi level energy (E_F_) and work function (ϕ). All data are in eV.

Structure	E_H_	E_L_	E_g_	E_F_	Φ
Tyramine	− 5.79	− 0.02	5.77	− 2.91	2.91
C_60_	− 5.98	− 3.23	2.75	− 4.61	4.61
BC_59_	− 5.66	− 3.22	2.44	− 4.44	4.44
SiC_59_	− 5.82	− 3.65	2.17	− 4.74	4.74
Tyramine@C_60_(a)	− 5.84	− 3.16	2.68	− 4.50	4.50
Tyramine@C_60_(b)	− 5.84	− 3.17	2.67	− 4.51	4.51
Tyramine@C_59_B(a)	− 4.81	− 2.75	2.06	− 3.78	3.78
Tyramine@C_59_B(b)	− 4.99	− 2.82	2.17	− 3.91	3.91
Tyramine@C_59_Si(a)	− 4.68	− 2.61	2.07	− 3.65	3.65
Tyramine@C_59_Si(b)	− 4.78	− 2.64	2.14	− 3.71	3.71

**Figure 2:**
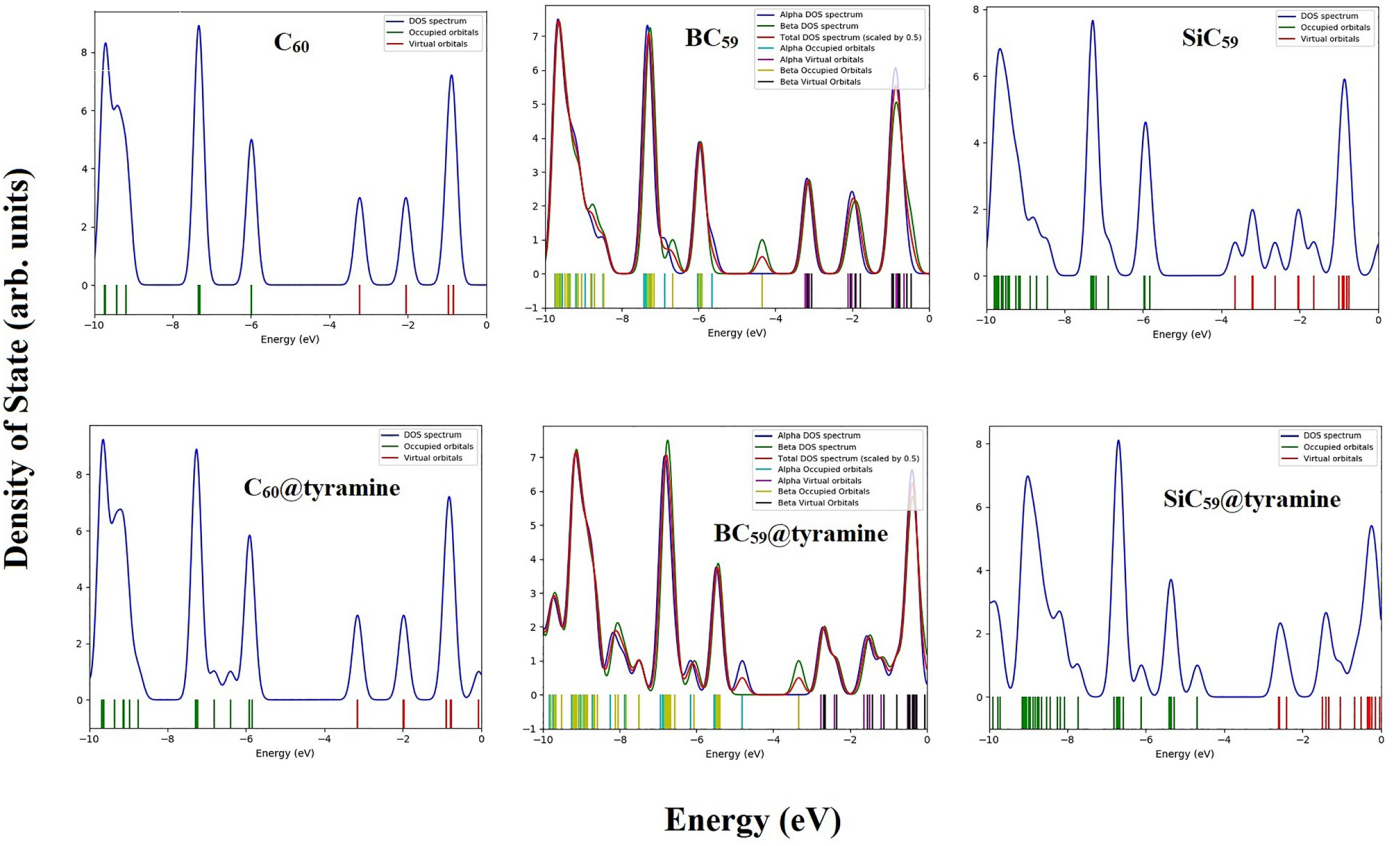
The density of states (DOS) for (**a**) C_60_ (**b**) C_59_B (**c**) C59Si (**d**) Tyramine@C_60_ (**e**) Tyramine@C_59_B (**f**) Tyramine@C_59_Si.

Some paramount electronic parameters viz HOMO energy $(\varepsilon_{HOMO} ),$ LUMO energy $(\varepsilon_{LUMO} )$, energy gap (*E*_*g*_) and Fermi level (*E*_*F*_) in pristine, B and Si doped fullerenes along with these parameters for the most favorable adsorption configurations of tyramine@C_60_, tyramine@C_59_B and tyramine@C_59_Si nano-hybrid systems have been estimated. The proper understanding of HOMO and LUMO is very crucial in quantum chemistry as these helps in exploring the electronic characteristics^72^. The iso-surfaces of HOMO and LUMO orbitals are shown in Fig. S1a and b of supplementary material. According to Table 2, the HOMO values $(\varepsilon_{HOMO} )$ of pristine, B and Si doped fullerenes are computed to be − 5.98, − 5.66, and − 5.82 eV respectively. Furthermore, the LUMO values $(\varepsilon_{LUMO} )$ are − 3.23, − 3.22, and − 3.65 eV, respectively before adsorption of tyramine. These values $(\varepsilon_{HOMO} and \varepsilon_{LUMO} )$ after tyramine adsorption over the surfaces of pristine, B and Si doped C_60_ fullerenes has been altered and computed to be − 5.84, − 4.81, − 4.68, − 3.16, − 2.75, and − 2.61 eV in their most favorable configurations respectively. We have also computed the energy gaps at DFT/PBE level which are tabulated in Table S1. This table shows that the magnitude of energy gap are 1.27, 1.02, and 1.12 eV for tyramine@C_60_(a), tyramine@C_59_B(a) and tyramine@C_59_Si(a) respectively. We have applied GaussSum software^73^ for total densities of states (DOS) to investigate the electronic structure of tyramine on the surfaces of pristine, B and Si doped C_60_ fullerenes. Figure 2 presents the population analysis at each orbital together with to a simple illustration of the characteristics of the chemical orbitals in a particular energy spectrum.

Likewise, the energies for Fermi level and work functions are computed in the present study in order to explore the effect of tyramine adsorption on these parameters. Fermi energy has been computed in accordance to the relation given below:

4 $$E_{F} = \varepsilon_{HOMO} + \frac{{(\varepsilon_{LUMO} - \varepsilon_{HOMO} )}}{2}$$

Table 2 infers that Fermi levels of pristine, B and Si doped C_60_ fullerenes after tyramine adsorption up-shifted which is attributed to the electronic charge transfer from tyramine to fullerene systems. The work function (Φ =  − $E_{F}$) that is known to be least amount of energy to put out an electron from Fermi level is very crucial for the devolvement and reign of electronic devices is computed to be 4.61, 4.44, and 4.74 eV, respectively in case of pristine, B and Si doped C_60_ fullerenes. However, for the most favorable configurations *i.e.* tyramine@C_60_(a), tyramine@C_59_B(a) and tyramine@C_59_Si(a) nano-hybrid systems this value gets lowered by 0.11, 0.66, and 1.09 eV. This change in work function may causes for the alteration of the field emission properties of C_60_, C_59_B and C_59_Si fullerenes.

The thermodynamics functions of any molecular species are vital as these helps to fully characterize the nature of interactions in equilibrium. Further, these functions are crucial during estimating the direction of chemical process in accordance to the second law of thermodynamics. This notable fact motivated the authors to calculate the total and vibrational contributions of various prominent thermodynamic quantities, notably thermal energy, specific heat, entropy, and zero-point vibrational energy (ZPVE). The total and vibrational contribution of tyramine@C_60_(a), tyramine@C_59_B(a) and tyramine@C_59_Si(a) systems are listed in Table 3. The interaction of ethylamine side chain of tyramine when interacts with pristine C_60_ fullerene surface results 368.587 kcal/mol thermal energy. The doping of B and Si atoms do not shows any significant change for the value of thermal energy. The vibrational motions majorly contribute to total value of thermal energy in the most favorable adsorption configurations that compared to electronic and rotational motions. Furthermore, the contribution of vibrational motions reveals more for specific heat in all the above cited nano-hybrid systems. However, the vibrational motions show contribution of 59, 55, and 56% in case of total entropy for tyramine@C_60_(a), tyramine@C_59_B(a) and tyramine@C_59_Si(a) nano-hybrid systems, respectively. The zero-point vibrational energy (ZPVE) pays an important role enthalpy of formation calculations so we have computed it for tyramine@C_60_(a), tyramine@C_59_B(a) and tyramine@C_59_Si(a) nano-hybrid systems. The corresponding values are 347.957, 348.266, and 345.818 kcal/mol, respectively. The Gibss free energy is estimated to be 6.935 kcal/mole in tyramine@C_60_(a) suggesting endothermic adsorption of tyramine on pristine C_60_ fullerene. However, it is − 15.327 and − 16.579 kcal/mole in case of tyramine@C_59_B(a) and tyramine@C_59_Si(a) nano-hybrid systems which infers exothermic adsorption of tyramine on doped fullerenes.

**Table 3:** Some standard thermodynamical parameters of Tyramine@C_60_(a), Tyramine@C_59_B(a) and Tyramine@C_59_Si(a).

Parameters	Tyramine@C_60_(a)	Tyramine@C_59_B(a)	Tyramine@C_59_Si(a)
Thermal energy (kcal/mol)	368.587(366.809)	368.075(366.297)	366.135(364.357)
Specific heat (CV) (cal/mol-K)	157.584(151.622)	156.619(150.657)	159.159(153.197)
Entropy (S) (cal/mol-K)	208.380(122.796)	191.490(104.695)	195.043(109.459)
ZPVE (kcal/mol)	347.957	348.266	345.818
Gibbs free energy (ΔG) (kcal/mol)	6.935	-15.327	-16.579

## Conclusion

The present investigation focuses on the applications of pristine, B, and Si atoms doped fullerenes as a biosensor to detect an important neurotransmitter tyramine by performing a detailed in-silico experimental study on various parameters such as adsorption energy, HOMO-LUMO energy, Fermi energy, work function, and thermodynamic functions. The important findings of the ongoing investigation are summarized below:

1. DFT calculations have been employed to explore the adsorption of tyramine neurotransmitter over pristine, Boron (B) and Silicon (Si) fullerenes.
2. The non-covalent interactions C–H···π, N–H···π and O–H···π interactions play crucial role in adsorption of tyramine over fullerenes *i.e.* C_60_, C_59_B, and C_59_Si fullerenes.
3. The adsorption of tyramine over pristine C_60_ fullerene is weak while doping of B or Si atoms enhanced the adsorption capacity of fullerenes.
4. A significant reduction in band gap of B and Si atom doped fullerenes has been noted which causes to change in electrical conductivity.
5. The direction of charge transfer is from tyramine to C_60_, C_59_B, and C_59_Si fullerenes that is 0.006, 0.394, and 0.257*e* in magnitude respectively.
6. The magnitude of Gibss free energy has been computed to be 6.935, − 15.327 and − 16.579 kcal/mole which showed that endothermic adsorption of tyramine on pristine C_60_ fullerene while exothermic adsorption of tyramine on doped fullerenes.

### Supplementary Information

Supplementary Information.

## Data Availability

All data generated or analyzed during this study are included in this published article and its supplementary information files.

## References

[1] Afreen S, Muthoosamy K, Manickam S, Hashim U (2015). Functionalized fullerene (C60) as a potential nanomediator in the fabrication of highly sensitive biosensors. Biosens. Bioelectron..

[2] Yeh CH, Hsiao YJ, Jiang JC (2019). Dopamine sensing by boron and nitrogen co-doped single-walled carbon nanotubes: A first-principles study. Appl. Surf. Sci..

[3] Li Y, Hodak M, Lu W, Bernholc J (2017). Selective sensing of ethylene and glucose using carbon-nanotube-based sensors: An ab initio investigation. Nanoscale.

[4] Bati ASR, Yu LP, Batmunkh M, Shapter JG (2019). Recent advances in applications of sorted single-walled carbon nanotubes. Adv. Funct. Mater..

[5] Shaalan NM, Saber O, Ahmed F, Alijaafari A, Kumar S (2021). Growth of defect-induced carbon nanotubes for low-temperature fruit monitoring sensor. Chemosensor.

[6] Asencio LP, Anota EC, Hernandez EG (2022). Insights on α-glucose biosensors/carriers based on boron-nitride nanomaterials from an atomistic and electronic point of view. ChemPhysChem..

[7] Miah MH, Hossain MR, Islam MS, Ferdous T, Ahmed F (2021). A theoretical study of allopurinol drug sensing by carbon and boron nitride nanostructures: DFT, QTAIM, RDG, NBO and PCM insights. RSC Adv..

[8] Sun Q, Zhang R, Qiu J, Liu R, Xu W (2018). On-surface synthesis of carbon nanostructures. Adv. Mater..

[9] Ergürhan O, Parlek C, Alver Ö, Senyel M (2018). Conformational and electronic properties of hydroquinone adsorption on C60 fullerenes: Doping atom, solvent and basis set effects. J. Mol. Struct..

[10] Vishwkarma AK, Yadav T, Pathak A, Brahmachari G (2023). Interaction of a synthetic bio-relevant drug-molecule with C24 and B12N12 fullerene: A first-principles quantum chemical investigation. Diam. Related Mater..

[11] Opoku F, Osikoya AO, Dikio ED, Govender PP (2019). One-step synthesized 2D heteroatom doped graphene for high throughput electrochemical biosensing: A combined experimental and computational studies. Diam. Related Mater..

[12] Fellah MF (2019). Pt doped (8,0) single wall carbon nanotube as hydrogen sensor: A density functional theory study. Int. J. Hydr. Ener..

[13] Shaalan NM, Ahmed F, Kumar S, Melaibari A, Hasan PMZ, Aljaafari A (2020). Monitoring food spoilage based on a defect-induced multiwall carbon nanotube sensor at room temperature: Preventing food waste. ACS Omega.

[14] Choi JH, Lee J, Byeon M, Hong TE, Park H, Lee CY (2020). Graphene-based gas sensors with high sensitivity and minimal sensor-to-sensor variation. ACS Appl. Nano Mater..

[15] Chakroborty S, Bharadwaj V, Sahoo SK (2021). Sensing and Biosensing with 2D Nanosheets Beyond Graphene.

[16] Nhat PVu, Si NT, Tram NTT, Duong LV, Nguyen MT (2020). Elucidating the binding mechanism of thione-containing mercaptopurine and thioguanine drugs to small gold clusters. J. Comput Chem..

[17] Panda P, Pal K, Chakroborty S (2021). Smart advancements of key challenges in graphene-assembly glucose sensor technologies: A mini review. Mat. Lett..

[18] Gao Y, Yu H, Tian J, Xiao B (2021). Nonenzymatic DNA-based fluorescence biosensor combining carbon dots and graphene oxide with target-induced DNA strand displacement for microRNA detection. Nanomaterial.

[19] Tiwari JN, Vij V, Kemp KC, Kim KS (2016). Engineered carbon-nanomaterial-based electrochemical sensors for biomolecules. ACS Nano..

[20] Liu Y, Dong X, Chen P (2012). Biological and chemical sensors based on graphene materials. Chem. Soc. Rev..

[21] Balasubramanian K, Burghard M (2006). Biosensors based on carbon nanotubes’. Anal. Bioanal. Chem..

[22] Ma Y, Yang J, Yang T, Deng Y, Gu M, Hu R, Yang Y (2020). Electrochemical detection of C-reactive protein using functionalized iridium nanoparticles/graphene oxide as a tag. RSC Adv..

[23] Bala K, Sharma D, Gupta N (2018). Carbon-nanotube-based materials for electrochemical sensing of the neurotransmitter dopamine. ChemElectroChem..

[24] Kruss S, Landry MP, Ende EV, Lima BMA, Reuel NF, Zhang J, Nelson J, Mu B, Hilmer A, Strano M (2014). Neurotransmitter detection using corona phase molecular recognition on fluorescent single-walled carbon nanotube sensors. J. Am. Chem. Soc..

[25] Kim B, Song HS, Jin HJ, Park EJ, Lee SH, Lee BY, Park TH, Hong S (2013). Highly selective and sensitive detection of neurotransmitters using receptor-modified single-walled carbon nanotube sensors. Nanotech..

[26] Nath N, Kumar A, Chakroborty S, Soren S, Barik A, Pal K, de Souza Jr FG (2023). Carbon nanostructure embedded novel sensor implementation for detection of aromatic volatile organic compounds: an organized review. ACS Omega.

[27] Nath, N., Chakroborty, S., Vishwakarma, D. P., Goga, G., Yadav, A. S., Mohan, R. A graphene-based material for green sustainable energy technology for hydrogen storage.* Environ. Sci. Poll. Res*. (1), 1–13 (2023). 10.1007/s11356-023-30431-w 37872339

[28] Gao S, Khan A, Nazari M, Mirzaei H, Lup ANK, Baei MT, Chandiramouli R, Soltani A, Salehi A, Javan M, Jokar MH, Pishnamazi M, Nouri A (2021). Molecular Modeling and Simulation of glycine functionalized B_12_N_12_ and B_16_N_16_ nanoclusters as potential inhibitors of proinflammatory cytokines. J. Mol. Liq..

[29] Cao Y, Khan A, Soltani A, Moghadam VE, Lup ANK, Aghaei M, Abdolahi N, Khalili M, Cordani M, Balakheyli H, Tavassoli S, Albadarin AB (2021). Spectroscopic, density functional theory, cytotoxicity and antioxidant activities of sulfasalazine and naproxen drugs combination. Arab. J. Chem..

[30] Hirsch A (2002). Functionalization of Single-Walled Carbon Nanotubes. Angew. Chem. Int. Ed..

[31] Luo P, Lu F, Chen L (2014). Recent advances on functional nucleic acid-based biosensors for detection of food contaminants, Anal. Chim. Acta..

[32] Wang YL, Su KH, Zhang JP (2012). Studying of B, N, S, Si and P Doped (5, 5) Carbon Nanotubes by the Density Functional Theory. Adv. Mater. Res..

[33] Hassani F, Tavakol H (2016). A DFT, AIM and NBO study of adsorption and chemical sensing of iodine by S-doped fullerenes. Sensor. Actuat. B-Chem..

[34] Amiraslanzadeh S (2016). The effect of doping different heteroatoms on the interaction and adsorption abilities of fullerene. Hetroatom Chem..

[35] Lo R, Manna D, Hobza P (2022). P-Doped graphene–C60 nanocomposite: A donor–acceptor complex with a P-C dative bond. Chem. Com..

[36] Yadav T, Mukherjee V (2017). Structural modeling and spectroscopic investigation of isolated and hydrochloride tyramine neurotransmitter. J. Mol. Struct..

[37] Makara K, Misawa K, Miyazaki M, Mitsuda H, Ishiuchi S, Fujii M (2008). Vibrational signature of the conformers in tyramine studied by IR dip and dispersed fluorescence spectroscopies. J. Phy. Chem. A..

[38] Lulinski P, Sobiech M, Zolek T, Maciejewska D (2004). A separation of tyramine on a 2-(4-methoxyphenyl)ethylamine imprinted polymer: An answer from theoretical and experimental studies. Talanta..

[39] Jimenez MG, Valganon JA, Santos MDPG, Calle E (2017). Casado, Mutagenic products are promoted in the nitrosation of tyramine. J. Food Chem..

[40] Kochana J, Wapiennik K, Knihnicki P, Pollap A, Janus P, Oszajca M, Kustrowski P (2016). Mesoporous carbon-containing voltammetric biosensor for determination of tyramine in food products. Anal. Bio. Chem..

[41] Chaniotakis N, Sofikiti N (2008). Novel semiconductor materials for the development of chemical sensors and biosensors: A review. Anal. Chim. Acta.

[42] Urdaneta I, Keller A, Atabek O, Palma JL, Shapiro DF, Tarakeshwar P, Mujica V, Calatayud M (2014). Dopamine adsorption on TiO_2_ anatase surfaces. J. Phys. Chem. C..

[43] Mu Q, Xu H, Li Y, Ma SJ, Zhong XH (2014). Adenosine capped QDs based fluorescent sensor for detection of dopamine with high selectivity and sensitivity. Analyst..

[44] Fang L, Huang K, Zhang B, Liu B, Zhang Q (2014). Nanosheet-based 3D hierarchical ZnO structure decorated with Au nanoparticles for enhanced electrochemical detection of dopamine. RSC Adv..

[45] Kim J, Rim YS, Chen H, Cao HH, Nakatsuka N, Hinton HL, Zhao C, Andrewa AM, Yang Y, Weiss PS (2015). Fabrication of high-performance ultrathin In_2_O_3_ film field-effect transistors and biosensors using chemical lift-off lithography. ACS Nano..

[46] Zhang X, Chen X, Kai S, Wang HY, Yang J, Wu FG, Chen Z (2015). Highly sensitive and selective detection of dopamine using one-pot synthesized highly photoluminescent silicon nanoparticles. Anal. Chem..

[47] Aarva A, Laurila T, Caro MA (2017). Doping as a means to probe the potential dependence of dopamine adsorption on carbon-based surfaces: A first-principles study. J. Chem. Phys..

[48] Pereira Silva AL, Silva ACA, Júnior JJGV (2022). Putrescine adsorption on pristine and Cu-decorated B12N12 nanocages: A density functional theory study. Comp. Theo. Chem..

[49] Shakerzadeh E (2021). Efficient carriers for anticancer 5-fluorouracil drug based on the bare and M−encapsulated (M = Na and Ca) B40 fullerenes; in silico investigation. J. Mol. Liq..

[50] Chatterjee M, Nath P, Kadian S, Kumar A, Kumar V, Roy P, Manik G, Satapathi S (2022). Highly sensitive and selective detection of dopamine with boron and sulfur co-doped graphene quantum dots. Sci. Rep..

[51] Mohammadi MD, Abdullah HY, Kalamse V, Chaudhari A (2022). Interaction of Fluorouracil drug with boron nitride nanotube, Al doped boron nitride nanotube and BC2N nanotube. A. Compt. Theor. Chem..

[52] Mohammadi MD, Abdullah HY, Biskos G, Bhowmick S (2021). Effect of Al- and Ga-doping on the adsorption of H_2_SiCl_2_ onto the outer surface of boron nitride nanotube: A DFT study. Compt. Rend. Chim..

[53] Kande EN, Abbasi M, Mohammadi MD (2018). DFT, QTAIM and NBO investigation of the interaction of rare gases with pristine and decorated boron nitride nanotube. Chem. Select..

[54] Kande EN, Abbasi M, Mohammadi MD (2019). Feasibility of pristine and decorated AlN and SiC nanotubes in sensing of noble gases: A DFT study. Chem. Select..

[55] Mohammadi MD, Abdullah HY (2022). Weak intermolecular interactions of cysteine on BNNT, BNAlNT and BC2NNT: A DFT investigation. Bull. Mater. Sci..

[56] Mohammadi MD, Abdullah HY (2022). Intermolecular Interactions between Serine and C60, C59Si, and C59Ge: A DFT study. Silicon.

[57] Mohammadi MD, Salih IH, Abdullah HY (2020). The adsorption of chlorofluoromethane on pristine and Ge-doped silicon carbide nanotube: a PBC-DFT, NBO, and QTAIM study. Mol. Simul..

[58] Mohammadi MD, Abdullah HY, Bhowmick S, Biskos G (2021). A comprehensive investigation of the intermolecular interactions between CH2N2 and X12Y12 (X = B, Al, Ga; Y = N, P, As) nanocages. Can. J. Chem..

[59] Mohammadi MD, Abdullah HY, Kalamse V, Chaudhari A (2022). Interaction of halomethane CH3Z (Z = F, Cl, Br) with X12Y12 (X = B, Al, Ga & Y = N, P, As) nanocages. Compt. Theor. Chem..

[60] Mohammadi MD, Abbas F, Louis H, Afahanam LE, Gber TE (2022). Intermolecular Interactions between Nitrosourea and Polyoxometalate compounds. Chem. Select..

[61] Rezvani M, Astaraki M, Rahmanzadeha A, Ganji MD (2021). Theoretical assessments on the interaction between amino acids and the g-Mg3N2 monolayer: Dispersion corrected DFT and DFT-MD simulations. Phys. Chem. Chem. Phys..

[62] Mostafavi M, Tanreh S, Astaraki M, Farjah B, Rasoolidanesh M, Rezvani M, Ganji MD (2022). Dispersion-corrected DFT investigations on the interaction of glycine amino acid with metal organic framework MOF-5. Phys. B.

[63] Sabet M, Tanreh S, Khosravi A, Astaraki M, Rezvani M, Ganji MD (2022). Theoretical assessment of the solvent effect on the functionalization of Au32 and C60 nanocages with fluorouracil drug. Diam. Relat. Mater..

[64] Aghahosseini A, Edjlali L, Jamehbozorgi S, Rezvani M, Ghasemi E (2023). Theoretical investigations of functionalization of graphene and ZnO monolayers with mercaptopurine at aqueous media: A dispersion-corrected DFT calculations and molecular dynamic simulations. J. Mol. Liq..

[65] Tanreh S, Rezvani M, Ganji MD (2023). Molecular simulation investigations on interaction properties of the teriflunomide–chitosan complex in aqueous solution. J. Phys. Chem. Sol..

[66] Yadav T, Shakerzadeh E, Vishwkarma AK, Singh PK, Pathak A, Chakroborty S, Pandey FP, Moharana S, Kumar R (2023). Histamine sensing by boron and silicon doped C_60_ fullerenes: A first principles investigation. Diam. Relat. Mater..

[67] M. J. Frisch, G. W. Trucks, H. B. Schlegel, G. E. Scuseria, M. A. Robb, J. R. Cheeseman, G. Scalmani, V. Barone, G. A. Petersson, H. Nakatsuji, X. Li, M. Caricato, A. V. Marenich, J. Bloino, B. G. Janesko, R. Gomperts, B. Mennucci, H. P. Hratchian, J. V. Ortiz, A. F. Izmaylov, J. L. Sonnenberg, D. Williams-Young, F. Ding, F. Lipparini, F. Egidi, J. Goings, B. Peng, A. Petrone, T. Henderson, D. Ranasinghe, V. G. Zakrzewski, J. Gao, N. Rega, G. Zheng, W. Liang, M. Hada, M. Ehara, K. Toyota, R. Fukuda, J. Hasegawa, M. Ishida, T. Nakajima, Y. Honda, O. Kitao, H. Nakai, T. Vreven, K. Throssell, J. A. Montgomery, Jr., J. E. Peralta, F. Ogliaro, M. J. Bearpark, J. J. Heyd, E. N. Brothers, K. N. Kudin, V. N. Staroverov, T. A. Keith, R. Kobayashi, J. Normand, K. Raghavachari, A. P. Rendell, J. C. Burant, S. S. Iyengar, J. Tomasi, M. Cossi, J. M. Millam, M. Klene, C. Adamo, R. Cammi, J. W. Ochterski, R. L. Martin, K. Morokuma, O. Farkas, J. B. Foresman, and D. J. Fox, Gaussian, Inc., Wallingford CT, *Gaussian16*, Revision C.01 (2016).

[68] Yadav P, Verma A, Sonker P, Sharma VP, Kumar A, Yadav T, Pal S, Saha S, Tewari AK (2022). The enthralling effect of packing on the light emission of pyridazinone based luminophore: Crystallographic, electronic absorption and computational studies. J. Mol. Struct..

[69] Yadav P, Verma A, Sharma VP, Singh R, Yadav T, Kumar R, Pal S, Gupta H, Saha S, Tewari AK (2022). The development of a robust folded scaffold as a fluorescent material using butylidine-linked pyridazinone-based systems via aromatic π⋯π stacking interactions. N. J. Chem..

[70] Ema SN, Khaleque MA, Ghosh A, Priya AA, Habiba U, Shamim SUD (2021). Surface adsorption of nitrosourea on pristine and doped (Al, Ga and In) boron nitride nanosheets as anticancer drug carriers: The DFT and COSMO insights. RSC Adv..

[71] Ahmadi A, Hadipour NL, Bagheri Z (2013). Effects of Al doping and double-antisite defect on the adsorption of HCN on a BC2N nanotube: Density functional theory studies. J. Phy. Chem. C..

[72] Yadav T, Mukherjee V (2020). Optimization of synephrine and its vibrational and electronic structures. Vib. Spect..

[73] Boyle NMO, Tenderholt AL, Langner KM (2008). cclib: A library for package-independent computational chemistry algorithms. J. Comput. Chem..

