# Supplementary material for: In silico investigation on sensing of tyramine by boron and silicon doped C60 fullerenes
Source: Sci Rep. 2023 Dec 14;13:22264. doi: 10.1038/s41598-023-49414-5 (PMC10721924; doi:10.1038/s41598-023-49414-5)
Supplement: Supplementary file 1 — Supplementary Information. [file 41598_2023_49414_MOESM1_ESM.docx]

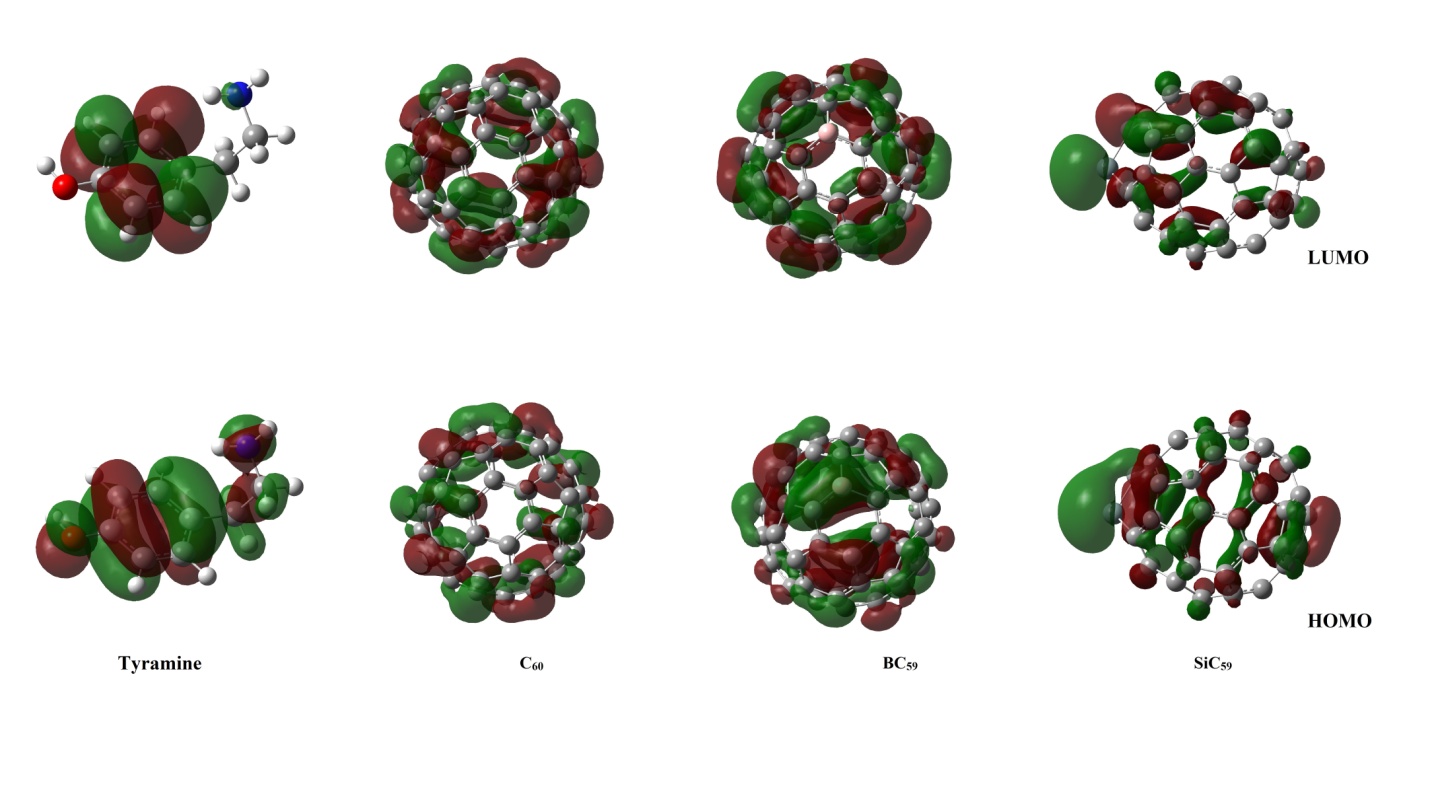


Fig.S1: HOMO and LUMO iso-surfaces of Tyramine, C60, C59B and C59Si


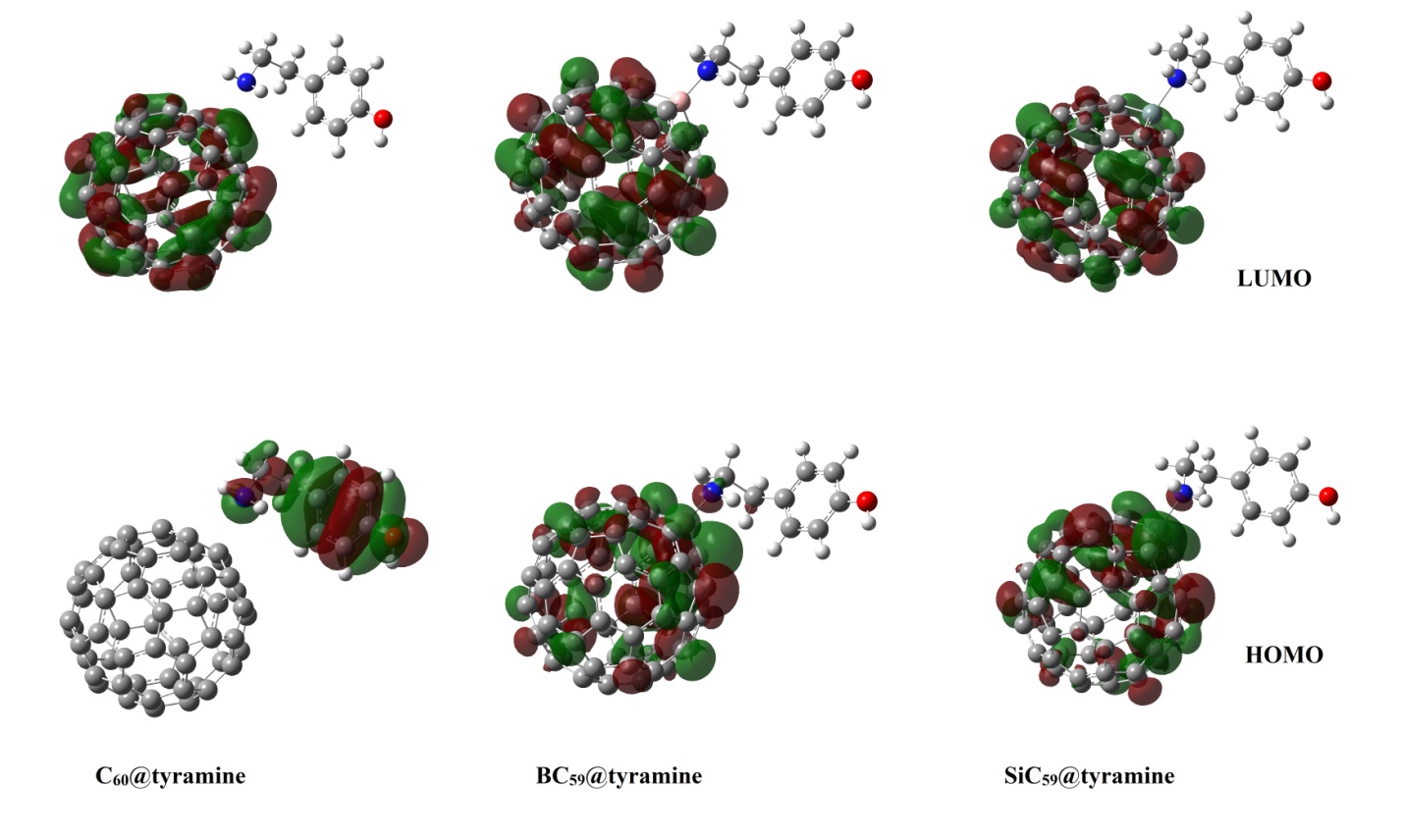


Fig.S2: HOMO and LUMO iso-surfaces of Tyramine@C60, Tyramine@C59B and Tyramine@C59Si

**Table-S1**: Calculated HOMO energy (E_H_), LUMO energy (E_L_), and HOMO-LUMO energy gap (E_g_) at DFT/PBE level. All data are in eV.

| **Structure** | **E_H_** | **E_L_** | **E_g_** |
| --- | --- | --- | --- |
| Tyramine | -5.65 | -0.24 | 5.41 |
| C_60_ | -5.49 | -3.78 | 1.71 |
| C_59_B | -5.15 | -3.78 | 1.37 |
| C_59_Si | -5.36 | -4.13 | 1.23 |
| Tyramine@C_60_(a) | -4.97 | -3.70 | 1.27 |
| Tyramine@C_60_(b) | -4.88 | -3.73 | 1.15 |
| Tyramine@C_59_B(a) | -4.31 | -3.29 | 1.02 |
| Tyramine@C_59_B(b) | -4.48 | -3.37 | 1.11 |
| Tyramine@C_59_Si(a) | -4.27 | -3.15 | 1.12 |
| Tyramine@C_59_Si(b) | -4.37 | -3.19 | 1.18 |

**Table-S2(a)**: Coordinates systems of studied systems

| **C_60_@tyramine(a)** | **C_60_@tyramine(b)** |
| --- | --- |
| Atom Coordinates (Angstroms)  X Y Z  C -2.129774 -0.522065 3.465623  C -0.803329 -1.085392 3.277149  C 0.278314 -0.247093 3.003459  C 0.078202 1.189145 2.906906  C -1.195438 1.730078 3.087957  C -2.321813 0.857134 3.372950  C -3.093001 -1.431540 2.868022  C -2.361926 -2.556937 2.310178  C -0.946814 -2.343075 2.563081  C -0.002878 -2.712394 1.603754  C 1.261321 -0.631722 2.004463  C 0.937603 1.692173 1.848386  C 0.489068 2.716036 1.012872  C -0.837279 3.279349 1.201425  C -1.662495 2.796511 2.218002  C -3.077606 2.582490 1.965153  C -3.485030 1.383906 2.678971  C -4.410038 0.510555 2.105266  C -4.210088 -0.925742 2.201715  C -2.776920 -3.131566 1.108099  C -3.940258 -2.604962 0.414193  C -4.642357 -1.524377 0.949907  C -5.109356 -0.458046 0.079811  C -4.965980 0.799618 0.793901  C -4.574550 1.950473 0.108364  C -3.611322 2.860036 0.705922  C -2.751900 3.362881 -0.352692  C -1.393061 3.568243 -0.109945  C 1.668705 0.566841 1.290529  C -0.094044 1.483655 -2.868874  C 1.023041 0.977857 -2.202567  C 1.222992 -0.458437 -2.106116  C 0.297985 -1.331788 -2.679824  C -0.865231 -0.805019 -3.373802  C -2.383717 1.137509 -3.278001  C -2.240230 2.395193 -2.563933  C -0.825121 2.609055 -2.311030  C -0.410128 3.183683 -1.108952  C 0.753213 2.657079 -0.415043  C 1.455311 1.576493 -0.950755  C 1.778933 -0.747501 -0.794749  C 1.387502 -1.898355 -0.109214  C 0.424274 -2.807920 -0.706772  C -0.109440 -2.530375 -1.966005  C -1.524549 -2.744395 -2.218855  C -1.991608 -1.677961 -3.088810  C -3.265247 -1.137026 -2.907755  C -3.465359 0.299211 -3.004309  C -3.184169 2.764514 -1.604604  C -4.310578 1.891468 -1.319618  C -4.448366 0.683839 -2.005312  C -4.855750 -0.514720 -1.291378  C -4.124647 -1.640055 -1.849237  C -3.676113 -2.663917 -1.013726  C -2.349767 -3.227233 -1.202276  C -1.793985 -3.516125 0.109090  C -0.435146 -3.310763 0.351839  C 1.922309 0.510166 -0.080662  C 1.123531 -1.839350 1.318768  C -1.057270 0.574182 -3.466473  C 7.369759 -0.613241 0.520264  C 8.440628 -1.333698 -0.031773  C 9.646540 -0.719825 -0.355570  C 9.808530 0.651363 -0.130327  C 8.757010 1.390348 0.418120  C 7.554368 0.758015 0.737803  H 8.330055 -2.401470 -0.206040  H 10.472802 -1.283927 -0.775540  H 8.877851 2.456402 0.600976  H 6.744164 1.342031 1.165667  C 6.051348 -1.288900 0.834058  H 6.234962 -2.316219 1.171312  H 5.548253 -0.762200 1.652162  C 5.086553 -1.338864 -0.364896  H 5.556921 -1.930079 -1.171327  H 4.182301 -1.880592 -0.062065  N 4.687389 0.011963 -0.768565  H 5.498584 0.504617 -1.136707  H 4.016192 -0.040456 -1.530994  O 11.014024 1.208217 -0.461599  H 10.993823 2.150095 -0.247556 | Atom Coordinates (Angstroms)  X Y Z  C 1.714426 2.220804 -2.776008  C 0.441816 1.567649 -3.033245  C -0.657788 1.836007 -2.216877  C-0.530212 2.768572 -1.109540  C0.691727 3.395805 -0.862576  C1.836806 3.116354 -1.712677  C2.773445 1.262379 -3.044292  C 2.155411 0.016878 -3.467303  C 0.714368 0.205531 -3.460560  C -0.123601 -0.834008 -3.054499  C -1.530444 0.753452 -1.794052  C-1.324105 2.262483 -0.002435  C-0.864352 2.403600 1.307632  C0.408154 3.056794 1.564806  C1.170444 3.542992 0.501759  C2.611489 3.354269 0.494862  C3.023265 3.090612 -0.873735  C 4.040247 2.170375 -1.131473  C 3.912843 1.237710 -2.238785  C2.701259 -1.203703 -3.067751  C 3.887841 -1.229495 -2.228984  C 4.481394 -0.033515 -1.822875  C 4.960070 0.113494 -0.458517  C4.687627 1.475626 -0.031232  C 4.292032 1.728656 1.282970  C 3.233010 2.687111 1.551347  C 2.439114 2.180827 2.658383  C 1.055385 2.361910 2.664930  C -1.942169 1.017014 -0.425391  C 0.110610 -1.186683 3.064349  C -1.028787 -1.162014 2.258842  C -1.156190 -2.094675 1.151531  C -0.139210 -3.014915 0.893795  C 1.047247 -3.040658 1.732734  C 2.442239 -1.491952 3.053303  C 2.169686 -0.129833 3.480619  C 0.728647 0.058821 3.487363  C 0.182799 1.279402 3.087810  C -1.003786 1.305193 2.249041  C -1.597338 0.109215 1.842930  C -1.803570 -1.399926 0.051288  C -1.407974 -1.652956 -1.262911  C -0.348953 -2.611412 -1.531290  C 0.272567 -3.278573 -0.474805  C 1.713610 -3.467296 -0.481701  C 2.192329 -3.320108 0.882635  C 3.414267 -2.692871 1.129597  C 3.541842 -1.760308 2.236934  C 3.007658 0.909710 3.074559  C 4.152777 0.630186 2.224387  C 4.414499 -0.677752 1.814109  C 4.826223 -0.941312 0.445450  C 4.208158 -2.186784 0.022494  C 3.748407 -2.327902 -1.287570  C 2.475903 -2.981096 -1.544748  C 1.828671 -2.286214 -2.644869  C 0.444941 -2.105129 -2.638323  C -2.076013 -0.037794 0.478577  C -1.268721 -0.554487 -2.204329  C 1.169628 -2.145106 2.796065  C-7.479718 0.161963 -0.610930  C-6.264764 0.863854 -0.568515  C-5.057237 0.222488 -0.310763  C -5.040145 -1.158160 -0.085709  C-6.236652 -1.878996 -0.122520  C -7.438870 -1.219458 -0.383427  H-6.263260 1.936474 -0.748010  H -4.120721 0.769876 -0.286304  H -6.230163 -2.954140 0.046398  H-8.362969 -1.790259 -0.411677  C-8.789965 0.880099 -0.857347  H-8.628709 1.712394 -1.553134  H-9.506579 0.197243 -1.326371  C -9.433874 1.439506 0.424552  H-8.747729 2.183775 0.867699  H-10.348645 1.977897 0.148550  N -9.808263 0.354451 1.335322  H-8.964904 -0.104338 1.673749  H-10.272312 0.737569 2.155339  O-3.827662 -1.743589 0.159648  H-3.957522 -2.692445 0.286790 |

**Table-S2(b)**: Coordinates systems of studied systems

| **BC_59_@tyramine(a)** | **BC_59_@tyramine(b)** |
| --- | --- |
| Atom Coordinates (Angstroms)  X Y Z  C -7.542235 -0.865992 -0.467907  C -8.422414 -1.158967 0.585666  C -9.406022 -0.261389 0.989122  C -9.531114 0.969344 0.335838  C -8.666604 1.284263 -0.715845  C -7.686088 0.371474 -1.108012  H -8.340037 -2.114844 1.097766  H -10.087721 -0.496989 1.799724  H -8.761598 2.237683 -1.232057  H -7.020630 0.626000 -1.928350  C -6.453987 -1.838562 -0.871652  H -6.813709 -2.866949 -0.745722  H -6.210173 -1.705914 -1.931261  C -5.155267 -1.677949 -0.060266  H -5.376876 -1.880319 1.003242  H -4.443461 -2.445908 -0.386484  N -4.546830 -0.367739 -0.305558  H -5.153186 0.358426 0.070329  H -3.668185 -0.295459 0.201698  O -10.516091 1.816304 0.766557  H -10.497722 2.618261 0.228149  C3.419361 2.992453 -0.730190  C2.753224 3.348808 0.512777  C 3.136025 2.736481 1.708997  C 4.190145 1.741399 1.711108  C 4.825201 1.394185 0.517284  C 4.431181 2.032465 -0.727728  C 2.423565 2.995771 -1.788510  C 1.145921 3.354501 -1.199446  C 1.349202 3.564733 0.223268  C 0.371002 3.150215 1.136079  C 2.125282 2.320229 2.666551  C 3.832201 0.708760 2.673130  C 4.126779 -0.630588 2.403367  C 4.781294 -0.990878 1.158604  C 5.120945 0.001642 0.233513  C 4.914108 -0.223384 -1.185551  C 4.486210 1.031315 -1.781642  C 3.527150 1.031656 -2.794353  C 2.476358 2.035384 -2.799527  C -0.027122 2.750948 -1.644620  C 0.027815 1.751380 -2.696914  C 1.253349 1.400295 -3.256637  C 1.545004 0.003159 -3.526037  C 2.949639 -0.223811 -3.248178  C 3.365020 -1.429063 -2.676125  C 4.364429 -1.428713 -1.626113  C 4.006468 -2.461142 -0.663857  C 4.215663 -2.247321 0.701400  C 2.559111 1.067708 3.265671  C 0.760956 -2.915298 1.814809  C 0.708220 -1.956040 2.824636  C -0.354702 -0.964377 2.827630  C -1.336182 -0.959041 1.830829  C -1.280171 -1.978171 0.757992  C 0.412224 -3.278389 -0.475057  C 1.819366 -3.498323 -0.196643  C 2.035827 -3.275571 1.222913  C 3.211485 -2.664281 1.663615  C 3.156528 -1.664432 2.716207  C 1.928228 -1.318351 3.283274  C 0.216024 0.290241 3.281709  C -0.197903 1.494293 2.703355  C -1.218163 1.484946 1.681471  C -1.835658 0.298200 1.299079  C -1.741906 -1.407668 -0.496688  C -1.043393 -1.694315 -1.665372  C 0.030459 -2.659761 -1.669679  C 2.788197 -3.099447 -1.121138  C 2.387985 -2.458568 -2.364152  C 1.038465 -2.235575 -2.623959  C 0.598048 -0.979915 -3.211760  C -0.678935 -0.623472 -2.630591  C -0.972075 0.735384 -2.389106  C -1.685081 1.138392 -1.201720  C -1.060195 2.338399 -0.701575  C -0.851896 2.522923 0.681649  C 1.622954 0.074290 3.564276  C 0.776993 2.520808 2.383153  C -0.247107 -2.921374 0.767443  B -2.117299 0.066370 -0.205955 | Atom Coordinates (Angstroms)  X Y Z  C -7.815937 0.156777 -0.628646  C -6.525314 0.701632 -0.519519  C -5.427681 -0.087843 -0.199132  C -5.599591 -1.460412 0.025056  C -6.874049 -2.023867 -0.078530  C -7.967191 -1.218281 -0.404138  H -6.374525 1.763323 -0.701619  H -4.430974 0.333859 -0.124922  H -7.015527 -3.089644 0.086175  H -8.950484 -1.672908 -0.494828  C -9.010202 1.034596 -0.945514  H -8.748359 1.770824 -1.714145  H -9.828074 0.431997 -1.348868  C -9.518106 1.825266 0.270679  H -8.729426 2.490542 0.636182  H -10.391082 2.429914 0.015206  N -9.924791 0.920840 1.398867  H -9.247208 0.153640 1.453459  H -9.839987 1.429056 2.280180  O -4.488431 -2.186700 0.336037  H -4.732931 -3.114654 0.450772  C 2.740064 1.982345 -2.715361  C 1.821938 2.905754 -2.065459  C 2.140509 3.436348 -0.812123  C 3.377806 3.059161 -0.155858  C 4.255107 2.167457 -0.776027  C 3.928485 1.618225 -2.081762  C 1.950023 0.978871 -3.407502  C 0.547795 1.283431 -3.182013  C 0.469635 2.470951 -2.351612  C -0.524499 2.561349 -1.365955  C 1.111291 3.549585 0.206520  C 3.115187 2.946883 1.271589  C 3.742358 1.947629 2.020943  C 4.650655 1.017482 1.373096  C 4.899887 1.124660 0.001902  C 4.973119 -0.069814 -0.820505  C 4.372822 0.233643 -2.109285  C 3.610696 -0.730591 -2.769367  C 2.376435 -0.349606 -3.434064  C -0.369597 0.253491 -2.995299  C 0.072786 -1.123741 -3.022198  C 1.415770 -1.420729 -3.235776  C 2.052141 -2.460995 -2.449566  C 3.408297 -2.038258 -2.163579  C 3.990583 -2.329578 -0.926749  C 4.785154 -1.327008 -0.243000  C 4.522693 -1.439898 1.184395  C 4.460131 -0.289944 1.976367  C 1.716054 3.253362 1.496218  C 1.110777 -0.890524 3.405826  C 0.685260 0.436090 3.432499  C -0.556258 0.811807 2.778580  C -1.338235 -0.145603 2.113350  C -0.889288 -1.548394 2.086023  C 1.219527 -2.821039 2.084567  C 2.579058 -2.395236 2.363922  C 2.512614 -1.199047 3.186007  C 3.435421 -0.168163 2.997615  C 2.991406 1.215865 3.025163  C 1.643460 1.510184 3.240189  C -0.364356 2.115502 2.182532  C -0.938375 2.391636 0.938574  C -1.760656 1.399911 0.283592  C -2.028664 0.176971 0.881966  C -1.280080 -2.169205 0.836517  C -0.355144 -2.983368 0.197239  C 0.898719 -3.334147 0.824955  C 3.565523 -2.510839 1.381590  C 3.230985 -3.056829 0.075229  C 1.920951 -3.449300 -0.195536  C 1.307432 -3.147717 -1.479161  C -0.093790 -2.857188 -1.262451  C -0.715304 -1.850742 -2.030225  C -1.696491 -0.962581 -1.461012  C -1.423606 0.350277 -1.987346  C -1.492955 1.502545 -1.176029  C 0.992282 2.550343 2.462291  C -0.185777 3.116902 -0.065123  C 0.311735 -1.894555 2.724666  B -2.259800 -1.173918 0.019328 |

**Table-S2(c)**: Coordinates systems of studied systems

| **SiC_59_@tyramine(a)** | **SiC_59_@tyramine(b)** |
| --- | --- |
| Atom Coordinates (Angstroms)  X Y Z  C -3.964718 -2.277627 -1.072622  C -3.136147 -3.092526 -0.198865  C -3.152992 -2.872441 1.178733  C -3.988985 -1.825857 1.740446  C -4.776708 -1.037370 0.900201  C -4.764981 -1.268274 -0.534954  C -3.221286 -2.038071 -2.296626  C -1.936669 -2.702821 -2.178354  C -1.877760 -3.345624 -0.879934  C -0.676660 -3.364282 -0.165599  C -1.911145 -2.906649 1.928633  C -3.261088 -1.214349 2.840793  C -3.352927 0.161849 3.058739  C -4.171402 0.982538 2.181613  C -4.869317 0.395139 1.124907  C -4.912256 1.050776 -0.171027  C -4.848842 0.023626 -1.197705  C -4.129040 0.253790 -2.371251  C -3.301691 -0.799418 -2.932060  C -0.788676 -2.121655 -2.704964  C -0.872359 -0.832486 -3.366314  C -2.099925 -0.187823 -3.468561  C -2.175486 1.240916 -3.232851  C -3.435370 1.517066 -2.563609  C -3.501951 2.503352 -1.579074  C -4.254695 2.267469 -0.359451  C -3.526821 2.879332 0.740715  C -3.488531 2.250853 1.987070  C -1.978122 -1.885119 2.960302  C 0.183917 2.125706 2.331951  C 0.264157 0.889578 2.966091  C 1.106110 -0.149829 2.410289  C 1.875202 0.064653 1.255949  C 1.789615 1.383141 0.579557  C 0.123011 3.186383 0.249477  C -1.135719 3.458009 0.925395  C -1.096829 2.802596 2.218721  C -2.248031 2.213084 2.741514  C -2.164190 0.921511 3.404098  C -0.932783 0.275442 3.515164  C 0.421742 -1.415646 2.610341  C 0.477555 -2.393965 1.620190  C 1.283061 -2.184909 0.435609  C 2.080332 -1.042024 0.331723  C 1.928585 1.295665 -0.867523  C 1.014084 1.958740 -1.690103  C 0.130270 2.956044 -1.124391  C -2.327378 3.495240 0.200151  C -2.311274 3.257413 -1.233560  C -1.105078 2.984405 -1.876483  C -1.021822 1.953024 -2.893401  C 0.274468 1.292446 -2.787836  C 0.350607 -0.092423 -3.064955  C 1.219409 -0.979052 -2.324239  C 0.486131 -2.180207 -1.993911  C 0.537797 -2.764187 -0.706762  C -0.836313 -1.154410 3.291587  C -0.707150 -3.145757 1.268320  C 0.943005 2.362832 1.121282  Si 2.577018 -0.375061 -1.318666  C 6.477821 -0.434614 0.665774  C 7.725596 -1.059713 0.817015  C 8.912698 -0.415073 0.483645  C 8.875194 0.890681 -0.017087  C 7.645201 1.534236 -0.176915  C 6.464104 0.872767 0.163463  H 7.769238 -2.072362 1.211186  H 9.874392 -0.901469 0.609575  H 7.608865 2.551941 -0.560927  H 5.513477 1.383534 0.036979  C 5.192522 -1.162505 0.999493  H 5.357291 -1.827063 1.856261  H 4.420693 -0.441533 1.289823  H 4.643344 -2.004509 -0.166804  H 5.386387 -2.782076 -0.420558  H 3.742388 -2.527967 0.175421  N 4.264976 -1.148300 -1.294082  H 5.101743 -0.719348 -1.684075  H 3.862867 -1.714629 -2.037136  O 10.069280 1.483401 -0.326734  H 9.900828 2.378192 -0.649733 | Atom Coordinates (Angstroms)  X Y Z  C -8.599946 0.201149 -0.631736  C -7.330349 0.793708 -0.718687  C -6.170881 0.089425 -0.409609  C -6.259309 -1.245178 0.000448  C -7.511885 -1.857304 0.095015  C -8.664685 -1.135965 -0.219806  H -7.247111 1.828646 -1.042274  H -5.192041 0.551361 -0.485652  H -7.587785 -2.896839 0.408249  H -9.633246 -1.622603 -0.144678  C -9.854906 0.991695 -0.937186  H -9.652677 1.706731 -1.743824  H -10.643373 0.317673 -1.289074  C -10.399656 1.770737 0.274077  H -9.637460 2.504606 0.592832  H -11.276833 2.345379 -0.047165  N -10.826626 0.853367 1.333860  H -10.011533 0.371852 1.708079  H -11.224935 1.381213 2.106699  O -5.090762 -1.896316 0.289513  H -5.293623 -2.804832 0.547976  C 3.756860 -0.675568 -2.835921  C 2.828118 0.312769 -3.360012  C 2.917401 1.640769 -2.941748  C 3.929578 2.035868 -1.977830  C 4.814560 1.084707 -1.467602  C 4.727017 -0.298534 -1.905899  C 3.031388 -1.921174 -2.661511  C 1.658025 -1.702007 -3.076193  C 1.527718 -0.320791 -3.497888  C 0.359314 0.389026 -3.207618  C 1.707189 2.388136 -2.654467  C 3.342146 3.030844 -1.094945  C 3.666206 3.036593 0.263292  C 4.585864 2.044052 0.794211  C 5.148878 1.088561 -0.053612  C 5.265631 -0.292139 0.383637  C 5.006087 -1.150365 -0.760569  C 4.303683 -2.344690 -0.591701  C 3.298955 -2.737895 -1.563387  C 0.608607 -2.310050 -2.396512  C 0.887086 -3.160079 -1.253603  C 2.201301 -3.360304 -0.846529  C 2.518478 -3.344986 0.568295  C 3.823859 -2.726626 0.726607  C 4.078652 -1.903831 1.824145  C 4.813797 -0.663121 1.651095  C 4.226441 0.331622 2.534296  C 4.117462 1.659180 2.115292  C1.969446 3.252149 -1.515583  C 0.485319 2.022073 2.660567  C 0.218298 2.837130 1.564681  C-0.798411 2.446784 0.609643  C -1.551498 1.273868 0.774372  C -1.266685 0.404505 1.943273  C0.666131 -0.197690 3.371486  C 1.969800 0.427958 3.526584  C 1.857008 1.806042 3.088483  C 2.908108 2.411581 2.399560  C 2.629108 3.263197 1.254520  C 1.311105 3.472355 0.848042  C -0.327975 2.836719 -0.708429  C -0.570982 2.007150 -1.800852  C -1.361660 0.805202 -1.639843  C -1.961220 0.529125 -0.408206  C -1.456245 -1.012262 1.664265  C -0.466571 -1.926967 2.033697  C 0.584699 -1.520449 2.942182  C 3.131684 -0.295462 3.254358  C 3.038719 -1.676218 2.810264  C 1.787479 -2.269088 2.649964  C 1.507930 -3.117009 1.506424  C 0.123416 -2.911025 1.095858  C -0.188159 -2.973949 -0.282335  C -1.171936 -2.106145 -0.889531  C -0.639151 -1.597341 -2.133254  C -0.752878 -0.236230 -2.500538  C 0.973450 3.469216 -0.562543  C 0.463273 1.772914 -2.784713  C -0.255639 0.790028 2.837234  Si -2.383912 -1.203183 0.077349 |
